# Involvement of the Cuneate Nucleus in the Acupuncture Inhibition of Drug-Seeking Behaviors

**DOI:** 10.3389/fnins.2019.00928

**Published:** 2019-08-29

**Authors:** Suchan Chang, Yeonhee Ryu, Yu Fan, Se Kyun Bang, Nam Jun Kim, Jin Gyeom Lee, Jin Mook Kim, Bong Hyo Lee, Chae Ha Yang, Hee Young Kim

**Affiliations:** ^1^College of Korean Medicine, Daegu Haany University, Daegu, South Korea; ^2^Korean Medicine Fundamental Research Division, Korea Institute of Oriental Medicine, Daejeon, South Korea; ^3^Korean Convergence Medicine, University of Science and Technology, Daejeon, South Korea

**Keywords:** acupuncture, dorsal column pathway, cuneate nucleus, morphine, ethanol, self-administration

## Abstract

Our previous studies have shown that acupuncture suppresses addictive behaviors induced by drugs of abuse, including cocaine, morphine and ethanol, by modulating GABA neurons in the ventral tegmental area (VTA) and dopamine (DA) release in the nucleus accumbens (NAc). The mechanisms by which the peripheral signals generated by acupoint stimulation are transmitted to brain reward systems are largely unexplored. The present study aims to investigate the role of spinal dorsal column (DC) somatosensory pathways in the acupuncture inhibition of drug addictive behaviors. Thus, we tested whether acupuncture at *Shenmen* (HT7) points reduces drug-seeking behaviors in rats self-administering morphine or ethanol and whether such effects are inhibited by the disruption of the cuneate nucleus (CN). The stimulation of HT7 suppressed morphine and ethanol self-administration, which were completely abolished by surgical lesioning of the CN. In *in vivo* extracellular recordings, single-unit activity of the CN was evoked during acupuncture stimulation. The results suggest that acupuncture suppresses morphine- and ethanol-seeking behaviors through the modulation of the CN, second-order neurons of the DC somatosensory pathway.

## Introduction

Over the last three decades, there has been an increasing interest in the treatment of substance abuse by acupuncture (Cui et al., 2013). We and others have demonstrated that acupuncture attenuates drug-seeking behaviors and relapse through the modulation of the mesolimbic dopamine (DA) system in animals and humans (Yoon et al., 2004, 2010, 2012; Yang et al., 2010). While the fundamental relationship between somatic input signals induced by acupuncture and brain reward systems are not largely understood, our previous studies have suggested peripheral and spinal mechanisms underlying the inhibitory effects of acupuncture on acute cocaine-induced locomotor activity. Our previous studies have shown that acupuncture at *Shenmen* (HT7) points activates peripheral sensory afferents and that acupuncture-initiated impulses in turn activate large A-fibers within the ulnar nerve trunk, resulting in the inhibition of acute cocaine-induced locomotion (Kim et al., 2013). Furthermore, our recent study revealed that the modulation of the dorsal column (DC) somatosensory pathway by acupuncture signals that stimulate HT7 suppresses cocaine-induced locomotor activity, an effect that is abolished by lesioning of the DC pathway including cuneate nucleus (CN, second order neurons of DC), but not to the spinothalamic tract (Chang et al., 2017). Acupuncture can suppress acute cocaine-induced locomotion through the spinal DC pathway (Kim et al., 2013; Chang et al., 2017). However, there is no direct evidence that acupuncture signals conveyed by the spinal DC pathway attenuate the reinforcing effects of drugs of abuse. Therefore, the present study extended prior work by testing the effects of acupuncture on drug-taking behaviors in rats self-administering morphine or ethanol. A role for the CN in the effects of acupuncture is also explored.

## Materials and Methods

### Subjects

Male Sprague-Dawley rats (*n* = 64; Daehan Animal, Korea) weighing 250–280 g at the beginning of the experiment were housed singly on a 12-hr light and dark cycle with free access to food and water. All procedures were approved by the Institutional Animal Care and Use Committee at Daegu Haany University and conducted in accordance with the National Institutes of Health guidelines for the care and use of laboratory animals (National Research Council, 2010).

### Acupuncture Treatment

**FIGURE 1:**
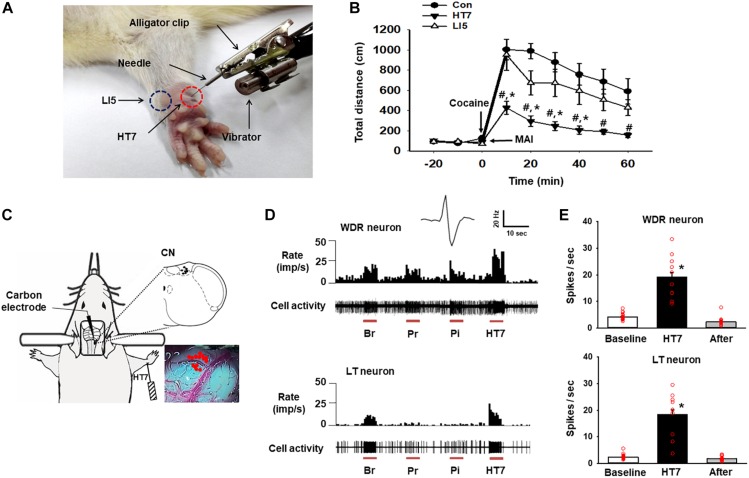
Activation of the cuneate nucleus by mechanical acupuncture at HT7. **(A)** Acupuncture treatment at HT7 (red circle) or LI5 (blue circle) was performed by using our MAI. A bar-type cell phone motor was attached to an alligator clip to generate mechanical vibration of the needle. The intensity, frequency and operation time were controlled by a motor controller. **(B)** The effect of MAI acupuncture at HT7 or LI5 on cocaine-induced locomotor activity. Con, cocaine only (*n* = 6); HT7, acupuncture at HT7 after cocaine injection (*n* = 6); LI5, acupuncture at LI5 after cocaine injection (*n* = 6). #*p* < 0.05 vs. Con, ^∗^*p* < 0.05 vs. LI5 by two-way repeated ANOVA, Tukey’s *post hoc* test, interaction *F* = 2.647, *p* = 0.011; normality test (Shapiro–Wilk) passed, *P* = 0.747. **(C)** A schematic drawing of the *in vivo* extracellular recordings of the CN (cuneate nucleus). **(D)** The neuronal activity of wide-dynamic-range (WDR) and low-threshold (LT) neurons in response to brush (Br), light pressure (Pr) and pinch (Pi) mechanical stimulation of the somatic field and acupuncture stimulation at HT7 (HT7). **(E)** The mean firing rates of WDR neurons (upper panel), (*n* = 12, ^∗^*p* < 0.05 vs. Baseline by one-way repeated ANOVA, Tukey’s *post hoc* test, *F*(2,38) = 74.171, *p* < 0.001; normality test (Shapiro–Wilk) passed, *P* = 0.727) and LT neurons (lower panel), (*n* = 15, ^∗^*p* < 0.05 vs. Baseline by one-way repeated ANOVA, Tukey’s *post hoc* test, *F*(2,38) = 76.851, *p* < 0.001, normality test (Shapiro–Wilk) passed, *P* = 0.317) over 10 s before (Baseline), during (HT7), and after (After) acupuncture at HT7.

Acupuncture was performed as described elsewhere (Kim et al., 2013). Briefly, while an assistant lightly restrained the rat, a needle (0.10 mm thick, 10 mm long; Dongbang Medical, South Korea) was bilaterally (except during the CN recordings) inserted 3 mm deep into the HT7 or LI5 acupoint, located on the transverse crease of the wrist of the forepaw, and stimulated for 20 sec (10 s for *in vivo* extracellular recordings) in duration and 1.3 m/sec^2^ in intensity by using a mechanical acupuncture instrument (MAI) that was developed by our laboratory (Kim et al., 2013; Figure 1A). The needle was maintained in place for up to 1 min after insertion and then withdrawn. The rats received acupuncture treatment only once.

### Cocaine-Induced Locomotor Activity

Locomotor activity was measured as previously described (Kim et al., 2013; Chang et al., 2017). Briefly, each animal was placed in an open field box and monitored with an image analysis system. After recording baseline activity for 30 min, the animal was given an intraperitoneal injection of cocaine (15 mg/kg) alone or in combination with acupuncture at HT7 or LI5 and monitored for up to 60 min after injection. The distance traveled was analyzed. The data are expressed as a percentage of the baseline activity.

### *In vivo* Extracellular Recording of the Cuneate Nucleus

Extracellular single-unit recordings of the CN were performed in 11 rats as previously described (Qin et al., 2010). In brief, under isoflurane anesthesia, a carbon-filament glass microelectrode (0.4–1.2 MΩ, Carbostar-1, Kation Scientific, United States) was stereotaxically inserted in the CN (0–0.8 mm deep from the dorsal surface of medulla, 1 mm caudal to the obex and 1–2 mm lateral from the midline) (Paxinos and Watson, 1998). Single-unit activity from the discharges was isolated, recorded and analyzed via a CED 1401 Micro3 device and Spike2 software (Cambridge Electronic Design, Cambridge, United Kingdom). After the basal single-unit neuronal activity of the CN neurons was recorded for 20 s prior to stimulation, we monitored single-unit activity following mechanical stimulation (10-sec duration) induced by brushing the receptive area, pressure stimulation (10-sec duration) and pinch stimulation (10-sec duration). The phenotypes of the cuneate neurons were classified according to previous studies (Qin et al., 2010) as follows: high-threshold (HT), wide-dynamic-range (WDR), and low-threshold (LT). The rats were then given MAI acupuncture at unilateral HT7 for 10 s, and the mean values of the firing rates in the 20 s before, 10 s during and 10 s after acupuncture were compared.

### Surgical Transection of the Cuneate Nucleus

Bilateral lesions of the CN were made as described elsewhere (McKenna and Whishaw, 1999; Wang and Thompson, 2008) with slight modifications. The animal was placed in a stereotaxic frame with its head tilted forward, the obex was exposed surgically and the CN was macerated along its length with a fine forcep under a microscope. Sham lesions were made using the same procedure (incision of skin, muscle and occipital bone) except the lesion was not created in CN. For histological confirmation of the lesions, all animals were sacrificed at the end of experiment and the brain stems, including the CN, were removed, postfixed in 4% paraformaldehyde and cryoprotected in 30% sucrose. The tissue was then cryosectioned into 30 μm-thick sections and stained with toluidine blue. Only the rats with confirmed CN lesions were included in the data analysis.

### Morphine Self-Administration Procedure

**FIGURE 2:**
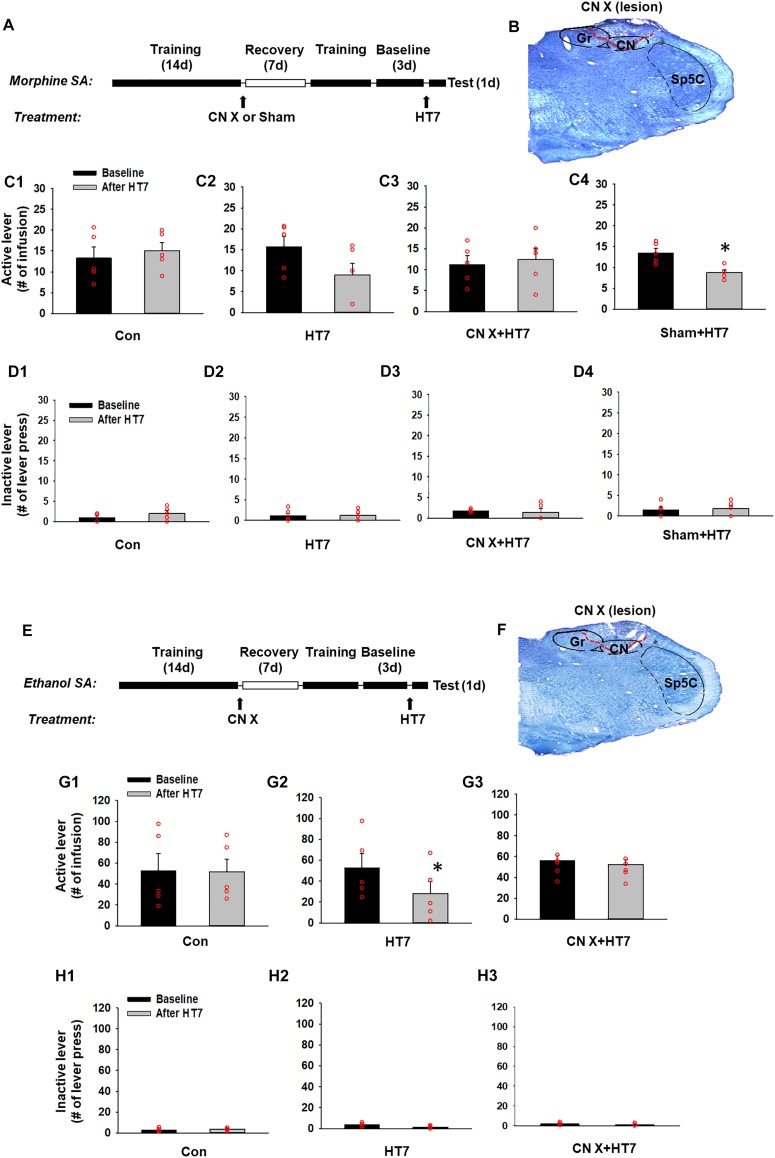
Effect of cuneate nucleus (CN) lesions on the inhibition of morphine or ethanol self-administration behaviors by acupuncture at HT7. **(A–D)** The effect of CN lesions on the inhibition of morphine self-administration behaviors by acupuncture at HT7. A schematic of the procedure of the morphine self-administration experiment **(A)**. While sham operation without HT7 acupuncture did not affect the number of active lever presses (Con) **(C1)**, the active lever response in the HT7-treated group was significantly reduced after HT7 stimulation compared to baseline (HT7; paired *t*-test, ^∗^*p* = 0.008 vs. Baseline) **(C2)**. These acupuncture effects were ablated in the rats with CN injury (CN X+HT7) **(C3)**. HT7 acupuncture significantly reduced morphine intake in sham group, compared to baseline (Sham+HT7; paired *t*-test, ^∗^*p* = 0.017 vs. Baseline) **(C4)**. There were no differences in the number of inactive lever presses among the groups or before and after HT7 **(D1–D4)**. A representative toluidine blue-stained image of a CN lesion **(B)**. **(E–H)** The effect of cuneate nucleus (CN) lesions on the inhibition of ethanol self-administration behaviors by acupuncture at HT7. A schematic of the procedure of the ethanol self-administration experiment **(E)**. The sham operation without HT7 acupuncture did not affect the number of active lever presses (Con) **(G1)**. The numbers of active lever presses in the HT7-treated group was significantly reduced after HT7 stimulation compared to baseline (HT7; paired *t*-test, ^∗^*p* = 0.042 vs. Baseline) **(G2)**. This effect was not seen in the rats with CN injury (CN X+HT7; **G3**). There were no differences in the number of inactive lever presses among the groups or before and after HT7 **(H1–H3)**. A representative toluidine blue-stained images of a CN lesion **(F)**.

Morphine self-administration was carried out in operant chambers (MED Associates Inc., Georgia, VT, United States) equipped with two response levers as described previously (Yoon et al., 2010). Briefly, a total of five rats were allowed to press a lever for the self-administration of morphine (0.5 mg/kg in 0.1 ml over 5 s, time-out period of 10 s) on a fixed ratio 1 (FR1) schedule in daily 1-h sessions for 6 days a week. Responding to the active lever caused 5 s of illumination of the cue light and 15 s of extinction of the house light. After the establishment of the baseline (defined as a mean value in three consecutive lever responses that varied less than 20%), the rats underwent procedures as shown in Figures 2A,E. The groups were assigned as follows; Con (control manipulation without the insertion of needles), HT7 (HT7 acupuncture), CN X+HT7 (HT7 acupuncture in CN lesioned rats) and Sham+HT7 (sham lesion and HT7 acupuncture).

### Ethanol Self-Administration Procedure

Another set of animals were trained to self-administer ethanol orally in operant chambers using a modified sucrose-fading procedure as previously described (Yang et al., 2010). In brief, rats were initially allowed to press a lever to receive a sucrose solution (20% w/v) on an FR1 schedule to facilitate the acquisition of ethanol self-administration. Following the establishment of a stable response, the sucrose concentration was gradually decreased to 0%, and the ethanol concentration was raised to 10%. After acquiring stable self-administration baselines (with the total varying less than 20% from the average of three consecutive sessions), the rats were exposed to acupuncture and CN lesioning using the same experimental procedure as described above. The groups were assigned as follows; Con (control manipulation), HT7 (HT7 acupuncture), and CN X+HT7 (HT7 acupuncture in CN lesioned rats).

### Data Analysis

Statistical analysis was carried out using SigmaStat 3.5 software (Systat Software Inc., United States). All data are presented as the mean ± SEM (standard error of the mean) and were analyzed by one or two-way repeated measures analysis of variance (ANOVA) followed by *post hoc* testing using the Tukey method. Statistical significance was considered at *p* < 0.05.

## Results

The systemic injection of cocaine increased locomotor activity, and the effect lasted for approximately 60 min from the peak magnitude at 10 min. Acupuncture at HT7, but not at LI5, attenuated the cocaine-induced enhancement of locomotor activity (Figures 1A,B). Thus, HT7 was used as the verum acupoint in the following experiments.

In total, 27 neurons in the CN were isolated and examined for responses to somatic stimuli. The activity of WDR neurons (*n* = 12 cells) in the CN increased during brushing (Br), light pressure (Pr) and noxious pinch (Pi) stimulation of the somatic field. LT neurons (*n* = 15 cells) were activated by hair movement or light pressure but not by noxious pinch (Figures 1C,D). However, we did not record any HT neurons. The WDR and LT neurons exhibited spontaneous basal firing rates of 4.16 ± 0.27 Hz and 2.44 ± 0.21 Hz, respectively. When an acupuncture needle was inserted into the HT7 acupoint and stimulated for 10 s with the MAI (Figure 1A), the firing rates of WDR and LT neurons increased to 19.21 ± 1.76 Hz and 18.42 ± 1.79 Hz, respectively, and they quickly returned to baseline levels after the termination of stimulation (*p* < 0.05, Figures 1D,E), indicating the excitation of the CN during acupuncture at HT7.

Next, to determine whether acupuncture suppresses morphine-seeking behaviors via the modulation of the CN, rats were divided into four groups: control manipulation (Con, *n* = 5), acupuncture treatment (HT7, *n* = 5), CN sham lesion/acupuncture treatment (Sham, *n* = 5), and CN lesion/acupuncture treatment (CN X+HT7, *n* = 5). The effect of acupuncture at HT7 with or without surgical lesioning of the CN was compared in the rats self-administering morphine. While control manipulation without acupuncture did not affect morphine self-administration (Con group in Figure 2C1), acupuncture at HT7 reduced the number of active lever presses (9.00 ± 2.76), but not inactive lever presses (Figures 2D1–D4), compared to baseline (15.80 ± 2.39) (paired *t*-test, *p* = 0.008; HT7 group in Figure 2C2). On the other hand, in the animals given bilateral CN lesions (Figure 2B), the inhibitory effects of acupuncture on morphine self-administration behaviors were attenuated compared to the corresponding baseline levels (CN X+HT7 group in Figure 2C3), whileas the group with the sham surgery (same surgery except CN lesion) showed inhibitory effects of acupuncture on morphine intake (Sham+HT7; *P* = 0.017, paired *t*-test; Figure 2C4).

The experiment was repeated in the rats self-administering ethanol. The rats were divided into three groups: control manipulation (Con, *n* = 5), acupuncture treatment (HT7, *n* = 5) and CN lesion/acupuncture treatment (CN X+HT7, *n* = 5; Figure 2F). While control manipulation without acupuncture did not affect morphine self-administration (Con group in Figure 2G1), Acupuncture stimulation of HT7 significantly suppressed active lever responses (28.00 ± 11.96), but not inactive lever responses (Figures 2H1–H3), compared to baseline (52.80 ± 13.50) (paired *t*-test, *p* = 0.04; HT7 group in Figure 2G2). This effect was abolished by the surgical dissection of the bilateral CN before acupuncture at HT7 (CN X+HT7 group in Figure 2G3). These results suggest that the CN is modulated during the acupuncture inhibition of the reinforcing effects of drugs.

## Discussion

We have shown that acupuncture at HT7 can suppress selectively morphine and ethanol self-administration, but not general consummatory behaviors, through GABA receptors in the ventral tegmental area (VTA) and DA release in the nucleus accumbens (NAc) induced by drugs of abuse (Kim et al., 2005; Yang et al., 2010; Yoon et al., 2010). Consistent with our previous studies, the present study showed that acupuncture at HT7 attenuated morphine and ethanol self-administration behaviors. Most importantly, the inhibition of drug-taking behaviors by acupuncture at HT7 was inhibited by the surgical lesioning of the CN, suggesting the involvement of sensory inputs to the CN in the effects of acupuncture. Previously, we reported that the dorsal column-medial lemniscus (DC-ML) pathway is involved in the inhibitory effects of acupuncture at HT7 on cocaine-induced locomotor activity (Chang et al., 2017). Mechanical stimulation of an acupuncture needle inserted into the HT7 acupoint attenuates cocaine-induced locomotor activity via A-fiber modulation of the ulnar nerve (Kim et al., 2013), which is reversed by surgical lesioning of the CN (Chang et al., 2017) and second-order neurons of the DC-ML pathway. The CN has also been reported to be anatomically connected to certain acupuncture points on the forelimb. For example, a neuronal tracing study revealed that, when cholera toxin subunit B, a retrograde tracer, is injected into the PC8 acupoint, which is approximately 5 mm from HT7 in rats, transganglionically labeled axonal terminals are found mainly in the CN and the medial part of the deep laminae of the spinal dorsal horn (Cui et al., 2013), suggesting that the CN can be excited by the stimulation of acupoints on the forelimb in rats. This finding was further confirmed by our previous and present data showing that the mechanical stimulation of HT7 activates both LT (A-fibers) and HT afferent fibers (Aδ and C fibers) (Kim et al., 2013) and excites WDR (responsive to both low- and high-threshold stimuli) and LT (responsive to LT stimuli) neurons in the CN during *in vivo* extracellular recordings. Moreover, the present study showed that reduction of morphine- or ethanol-seeking behaviors by acupuncture at HT7 was ablated by surgical CN lesioning. This finding suggests the involvement of CN neurons in the effects of acupuncture on the reinforcing effects of drugs of abuse. However, one of the limitations of this study is that the electrophysiological recordings of WDR and LT neurons were conducted in anesthetized rats, whereas the behavioral studies were carried out in freely moving rats. It is possible that the state of the rats during the electrophysiological recordings may not accurately reflect the state of the rats during behavioral testing.

While the CN may be particularly important for the acupuncture inhibition of drug-taking behaviors, the exact mechanism by which afferent inputs from the CN function in the mesolimbic DA system to influence addictive behaviors remains elusive, but some evidence suggests that the mechanism is dependent on the lateral habenula (LHb). Our recent study revealed that acupuncture at HT7 activates LHb neurons that project to the VTA and that the electrolytic lesioning of the LHb reduces acupuncture inhibition of cocaine-induced locomotion (Chang et al., 2017). The LHb is known to convey inhibitory reward signals to the VTA that inhibit DA release in the NAc (Velasquez et al., 2014). Thus, the results suggest that the effects of acupuncture on morphine- and ethanol-seeking behaviors might result from the activation of CN inputs to the LHb-VTA/RMTg pathway via direct or indirect projections. Although the LHb is thought to be sensitive to somatosensory inputs (Benabid and Jeaugey, 1989; Gao et al., 1996), it is not known yet how these inputs enter the LHb. It has been shown that the LHb receives input from various structures, such as the lateral hypothalamus, entopeduncular nucleus and prefrontal cortex (Shelton et al., 2012). As none of these structures provide direct sensory inputs (projections) to the LHb, neural connections that conduct impulses from the CN to the LHb may require multisynaptic pathways. Determining the specific neural circuits between the CN and LHb that are involved in the acupuncture-mediated inhibition of drug-taking behaviors will require additional study.

In conclusion, the results suggest that the CN is involved in the effects of acupuncture on drug-seeking behaviors in rats.

## Ethics Statement

Male Sprague-Dawley rats (Daehan Animal, Korea) weighing 250–280 g at the beginning of the experiment, were housed on a 12 h light and dark cycle and freely accessed to food and water. All procedures were approved by the Institutional Animal Care and Use Committee at the Daegu Haany University and conducted in accordance with the National Institutes of Health guidelines for the care and use of laboratory animals.

## Author Contributions

HK and CY designed the experiments. SC, YR, SB, YF, NK, JL, JK, and BL conducted the experiments. HK was responsible for the overall direction of the project and for edits to the manuscript.

## Conflict of Interest Statement

The authors declare that the research was conducted in the absence of any commercial or financial relationships that could be construed as a potential conflict of interest.
